# Safety and efficacy of combined acetabular reconstruction and microwave ablation in the treatment of periacetabular metastatic disease: a retrospective clinical evaluation

**DOI:** 10.3389/fonc.2024.1484876

**Published:** 2024-10-31

**Authors:** Chuanxi Zheng, Jin Qiu, Xiayi Zhou, Gang Xu, Tao Lan, Shiquan Zhang, Wei Li

**Affiliations:** ^1^ Department of Musculoskeletal Oncology, The First Affiliated Hospital of Shenzhen University, Shenzhen Second People’s Hospital, Shenzhen, China; ^2^ Department of Orthopedics, National Cancer Center/National Clinical Research Center for Cancer/Cancer Hospital & Shenzhen Hospital, Chinese Academy of Medical Sciences and Peking Union Medical College, Shenzhen, China; ^3^ Department of Spine Surgery, Shenzhen Second People’s Hospital, The First Affiliated Hospital of Shenzhen University, Health Science Center, Shenzhen, China

**Keywords:** acetabular metastasis, microwave ablation, reconstruction, surgery, metastasis

## Abstract

**Introduction:**

The periacetabular bone defects caused by metastatic disease often necessitate acetabular reconstruction and various techniques have been employed with varying degrees of success. The purpose of this study was to evaluate the efficacy and safety of acetabular reconstruction in conjunction with adjuvant microwave ablation as a surgical intervention for patients with periacetabular metastases.

**Methods:**

Between January 2019 and September 2023, 17 consecutive patients with different tumor subtypes required surgical intervention for periacetabular metastases. The acetabular reconstruction was performed by utilizing an acetabular reconstructive cage and cement total hip arthroplasty with microwave ablation. A retrospective review was performed to assess pain relief, intraoperative details and postoperative complications. Functional status following procedures was determined by the 1993 Musculoskeletal Tumor Society (MSTS) score and the overall survival of patients was estimated by Kaplan-Meier analysis

**Results:**

In total, 8 males and 9 females were included with an average age of 48.6 years (range 34-66). Patients reported a significant improvement in the level of pain and the mean VAS score declined from 7.7 preoperatively to 2.2 postoperatively. Of the 17 patients, 16 could ambulate either independently (6 patients) or using a walking aid (10 patients) with a mean MSTS score of 18.9. The median follow‐up was 9.0 months. Nine patients were alive at the most recent follow-up with overall survival of 40.9% at 12 months and 30.7% at 36 months, respectively.

**Conclusion:**

In selected patients with periacetabular metastasis, the utilization of an acetabular cage and cement total hip arthroplasty presents a less invasive reconstruction technique. The incorporation of adjuvant microwave ablation has shown promise in providing long-lasting pain relief, reducing intraoperative bleeding, and improving local tumor control. However, further research and extended follow-up are necessary to establish the effectiveness of this procedure.

## 1 Introduction

In patients with advanced cancer, bone metastases commonly involve the pelvis, which is the second most common site for surgical intervention following the spine (1). Periacetabular bone destruction, often a result of mechanical forces transmitted from the trunk to the limbs, frequently leads to pelvic ring instability and pathological fractures. These conditions are associated with significant pain. These conditions are associated with significant pain, functional limitations, and decreased mobility. The primary treatment typically involves nonoperative measures, such as bone-modifying agents, chemotherapy, and radiotherapy, depending on the type of primary cancer and the extent of the lesions (2). Surgical intervention is indicated when nonoperative methods fail to control severe pain or in the presence of an impending or established pathological fracture (3).

Surgical management of periacetabular metastasis bone destruction is complex, with approaches varying based on the diversity of acetabular defects (4). In the past decade, a range of reconstructive methods has been described, from simple intralesional curettage to extensive resections with complex reconstructions. However, no consensus on the optimal approach has been established (5–7). Historically, Harrington’s technique was the mainstay, but it has been supplemented by advancements in cement-rebar reinforced hip reconstructions for total hip arthroplasty, including reinforced cemented cups and specialized acetabular components (8). While these have enhanced pain management and short-term outcomes, long-term follow-up reveals significant surgical trauma and postoperative complications (9). In addition to traditional Harrington procedures, antiprotrusio acetabular cages offer a promising solution for durable implant stability, effectively addressing severe bone destruction and even pelvic discontinuity (10). This procedure can be performed by using the commonly employed posterolateral approach, which is well-known among surgeons and has been shown to greatly enhance quality of life with a low incidence of complications (11). Nevertheless, recurrence of the disease at surgical sites is an unavoidable outcome following the palliative surgery involving intralesional curettage. It has been reported that local recurrence occurs in up to 35% of patients and often accompanied by intractable pain and implant failure (7).

In recent years, the advent of novel adjuvant therapies has significantly prolonged the survival of patients with metastatic tumors. As a result, the surgical management of bone metastases should address not only symptom palliation and functional improvement but also local tumor control throughout the extended survival period of the patient (12). However, management of periacetabular metastasis through simple intralesional curettage is often insufficient for long-term disease control, particularly in patients with renal cell carcinoma and thyroid cancer (13). Some studies have indicated that extensive resection of periacetabular metastases, classified as Harrington class III lesions, may offer a chance of cure in specific patient populations (14, 15). Nevertheless, such extensive resections require advanced reconstruction techniques to restore the iliofemoral weight-bearing axis, including custom or modular hemi-pelvic prostheses, reverse ice-cream cone prostheses, and pedestal cup fixation (14–16). It is noteworthy that these surgical procedures involve extensive soft tissue dissection, prolonged surgical time, and considerable blood loss (6, 7). Furthermore, the complexity of these reconstructions can compromise limb function, potentially offsetting the advantages of enhanced tumor control (7).

Microwave ablation (MWA) has emerged as a prominent technique in oncological treatment (17). Compared to other ablative methods, MWA offers the advantage of producing larger ablation volumes in less time, inducing coagulation necrosis and thereby causing cellular death within tumors (18, 19). The higher intralesional temperatures generated by MWA not only lead to tumor destruction but also impair tumor vasculature and perfusion (20). Consequently, MWA has been recognized as a safe and effective treatment for both primary bone tumors and metastatic lesions (21, 22). However, the combination of MWA with reconstruction surgery for periacetabular metastasis has not been investigated. Therefore, the objective of this study was to determine whether the use of acetabular reconstruction combined with adjuvant MWA is a safe and effective approach for periacetabular metastasis with minimal complication risks. We specifically sought to determine whether this combined approach could significantly alleviate pain, enhance functional outcomes, and effectively control local disease progression at the surgical sites.

## 2 Patients and methods

### 2.1 Patients selection

Following institutional review board approval, we conducted a retrospective review of the medical records of patients with metastatic disease in the periacetabular region. These patients were treated by utilizing an acetabular reconstructive cage and cement total hip arthroplasty with adjuvant MWA at our institution from January 2019 to September 2023. Due to the retrospective nature of this study, the requirement for informed consent was waived.

The inclusion criteria were defined as follows: (1) patients with symptomatic metastasis involving the periacetabular region; (2) the acetabular defects were classified as Harrington class II or III according to Harrington classification; (3) patients underwent acetabular reconstruction using acetabular reconstructive cages and cement total hip arthroplasty in conjunction with adjuvant microwave ablation; (4) the clinical records and radiological images of patients were available from electronic medical records. Patients with extensive acetabulum lesions and the life expectancy expected to be less than six weeks were excluded. Preoperatively, pelvic radiographs, computed tomographic (CT) scans, and magnetic resonance imaging (MRI), single-photon emission CT were used to assess the extent of periacetabular metastatic lesions and distribution of additional sites of bony disease. Next, the feasibility of periacetabular reconstruction using an acetabular cage was discussed by a multidisciplinary team. Extensive bone destruction was defined as the inferior and medial aspects of the acetabulum being destroyed according to the CT scans which were more appropriate for reconstruction with a hemi-pelvic implant. Patient demographic characteristics including age, gender, histologic type of primary tumor, previous or ongoing systemic anti-cancer therapy (chemotherapy, targeted therapy, hormone therapy and immunotherapy) or local radiotherapy, American Society of Anesthesiologists (ASA) and Eastern Cooperative Oncology Group (ECOG) score were collected from the electronic medical record.

### 2.2 Surgical technique

The indications for surgery include patients who experienced intractable pain despite conservative treatment, chemotherapy, and/or radiotherapy; patients with an impending or established pathological fracture affecting ambulation; the primary tumor was stable after sensitive systemic treatment, and life expectancy was expected to be more than six weeks. Preoperatively, two patients with renal cell carcinoma received selective arterial embolization to mitigate intraoperative hemorrhage.

All reconstructions were approached in a similar fashion and were performed by the same group of orthopedic surgeons. Patients were placed in a lateral decubitus position under general anesthesia, and a preoperative prophylactic antibiotic was used. A routine hip posterior-lateral approach with proximal extension was used to expose the involved hip and acetabulum. Then, the hip was dislocated followed by the resection of the femoral neck and the acetabulum was exposed after releasing the attachments of the rectus femoris and gluteal muscles. Once the acetabulum was adequately exposed, the gauzes soaked with cooling saline were utilized to isolate the tumor from the surrounding soft tissue prior to the ablation, especially for protection of the sciatic nerve. The ablation procedures were performed by using an MWA system (2450 MHz, MTI-5A, Great Wall, Nanjing, China), and the antenna of MWA was inserted into the metastatic lesion. Concurrently, a thermometer needle was strategically placed within the healthy tissue to monitor the temperature of the surrounding tissue during ablation, ensuring the protection of neurovascular structures. To achieve effective tumor cell inactivation, we maintained the core temperature of the lesions at 60-80°C by applying a power setting of 30-50W for a duration of 3-5 minutes, depending on the size of the lesion. An effective ablation margin of 2 cm was targeted, with microwave antennas repositioned to create overlapping zones for a larger margin, as determined by preoperative MRI. Additionally, cryogenic saline was utilized to cool adjacent normal tissue, preventing thermal damage to vital neurovascular structures.

Following ablation, the necrotic tissues, characterized by their black and friable appearance, were meticulously curetted to ensure complete removal. The acetabulum was prepared by reaming to facilitate the insertion of a reconstructive cage, which was sized 2 mm larger than the final reamer to ensure optimal inclination and anteversion. The acetabulum medial wall X-Change reconstructive cage (Stryker, Mahwah, NJ, USA) was then fixed with screws through the cage holes into the ischium and ilium. Vacuum-mixed polymethylmethacrylate (PMMA) cement was applied to fill any remaining acetabular defects using a cement gun. Digital pressure was applied to the cement within the residual defects, and a cement pressurizer was utilized to ensure thorough cement penetration into the adjacent bony defects. A Trident constraint acetabular cup (Stryker, Mahwah, NJ, USA) was then cemented into the cage with accurate inclination and anteversion. The femoral component was implanted in a standard fashion. Finally, imaging was promptly performed postoperatively to evaluate the implant positioning and acetabular reconstruction quality.

### 2.3 Postoperative management

All patients were allowed postoperatively fully weight-bearing ambulation with or without walking assistance and underwent rehabilitation as for standard total hip arthroplasty. The postoperative systemic and local therapy therapies for patients were decided by the multidisciplinary team, including chemotherapy, endocrinal therapy, targeted therapy, and local radiotherapy. The follow-up was requested monthly in the outpatient clinic over the first three months, and every three months thereafter for the outcome assessment. Both clinical and radiological evaluations were performed at each follow-up visit to assess the functional outcomes and local disease control. The Visual Analog Scale (VAS) was used to rate pain perception before the treatment and at the follow-up examination. The postoperative limb function was determined according to the Musculoskeletal Tumor Society (MSTS) score system, including six items: pain, overall function, emotional acceptance, support, walking ability, and gait (23). Moreover, postoperative complications such as wound infection, dislocation, aseptic loosening, periprosthetic fracture and local recurrence were also recorded. Follow-up duration was determined from the date of diagnosis until the date of death or last follow-up. The overall survival was estimated to describe the outcome of patients which was defined as the time from surgery to the death from any cause or was censored at the most recent follow-up.

### 2.4 Statistical analysis

The statistical analysis of this study was performed using STATA Statistical Software (version 16, College Station, TX, USA). Descriptive statistics including mean and standard deviation or range were calculated for continuous variables and categorical variables (number and percentage). A chi-square test (or Fisher exact test) was used to analyze the association between categorical variables, and an unpaired Student t-test was performed to compare continuous variables. Survival estimates were calculated using the Kaplan-Meier method. P-values with two-tailed significances of 0.05 were considered statistically significant.

## 3 Results

### 3.1 Patients’ characteristics

From January 2019 to September 2023, a total of 17 patients with metastatic lesions involving the periacetabular region were identified in this retrospective study. The baseline demographics and clinical characteristics of patients are presented in **Table 1**. The mean age of the patients at the time of surgery was 48.6 (range 34-66) years, including 8 males and 9 females. The most common primary malignancy was lung cancer (n=5, 29.4%) followed by colon carcinoma (n=2, 11.8%), cervical cancer (n=2, 11.8%) and renal carcinoma (n=2, 11.8%). Other primary malignancies included bladder (5.9%), gastrointestinal (5.9%), liver (5.9%), penile (5.9%), prostate (5.9%) and thyroid cancer (5.9%). Among them, 2 patients had displaced pathological fractures identified by CT scans and the remaining patients had impending fractures or intractable pain. There were 10 patients (58.8%) with Harrington class II lesions and 7 patients 41.2%) with Harrington class III lesions. ECOG 0-2 score was noted in 2 patients (11.8%) and 3-4 score in 15 patients (88.2%). The mean ASA physical status classification was 3 (range 2 to 3), including 7 patients scored 2 and 10 patients scored 3. Most of the patients had been heavily pretreated for their primary malignancy, 10 patients were treated with systemic therapy (including chemotherapy, endocrine therapy, targeted therapy, and immunotherapy) and 5 patients received preoperative radiotherapy, but 6 patients had not received any treatments prior to the surgical procedure. Of the17 periacetabular metastasis patients, 4 patients (23.5%) were affected by a single bone metastatic lesion, 13 patients (76.5%) presented multiple bone metastatic lesions and 8 patients (47.1%) were affected by concomitant visceral metastases (including lung, brain, liver and lymph nodes).

**Table 1:** Patient demographics and clinical baseline characteristics.

Characteristics		Patients (%)
Age		
	Mean	48.6
	Range	34-66
Gender		
	Female	9 (52.9%)
	Male	8 (47.1%)
Primary tumor		
	Bladder cancer	1 (5.9%)
	Cervical cancer	2 (11.8%)
	Colon carcinoma	2 (11.8%)
	Gastrointestinal cancer	1(5.9%)
	Liver cancer	1 (5.9%)
	Penile cancer	1(5.9%)
	Prostate cancer	1 (5.9%)
	Renal carcinoma	2 (11.8%)
	Thyroid cancer	1(5.9%)
	Lung cancer	5 (29.4%)
Harrington class		
	II	10 (58.8%)
	III	7 (41.2%)
Surgical indication		
	Intractable pain	6 (35.3%)
	Impending fracture	9 (52.9%)
	Pathologic fracture	2 (11.8%)
ECOG performance status		
	0-2	2 (11.8%)
	3-4	15 (88.2%)
ASA physical status		
	2	7 (41.2%)
	3	10 (58.8%)
Previous treatment		
	Systematic therapy	10 (58.8%)
	Radiotherapy	5 (29.4%)
	No	6 (35.3%)
Site of metastatic lesions		
	Solitary bone	4 (23.5%)
	Multiple bone	13 (76.5%)
	Visceral metastasis	8 (47.1%)

ECOG, Eastern Cooperative Oncology Group; ASA, American Society of Anesthesiologist.

### 3.2 Clinical and functional outcomes

The surgical indications for periacetabular metastasis were pathological fracture of the acetabulum in 2 patients (11.8%) or an osteolytic lesion at high risk for pathological fracture of the acetabulum in 9 patients (52.9%). In the remaining 6 patients (35.3%), surgical treatment was performed owing to a painful and progressive lesion that failed to respond to initial nonoperative management (**Table 2**). Eventually, all patients underwent intralesional curettage followed by adjuvant MWA and reconstruction using the acetabular reconstructive cage and cement total hip arthroplasty. The mean operative duration was 3.1 ± 0.1 hours (ranging from 2.1 to 4.0 hours). All patients, except 2 patients with renal metastasis, underwent selective arterial embolization before surgery to minimize intraoperative bleeding and the mean intraoperative hemorrhage was 358.8 ± 29.8 mL (range 200 to 600 mL). The mean preoperative VAS pain score was 7.7 ± 0.3 points. Among these patients, 2 were bed-bound, 5 were wheelchair-bound, and 10 required assistances for ambulation with a walker or double crutch. These patients experienced significant pain relief and functional improvement one month postoperatively, with a mean postoperative VAS of 2.2 ± 0.2 points, and this improvement was statistically significant (*p*< 0.01). Subjectively, 15 of 16 patients (93.8%) reported reduced pain levels postoperatively. Specifically, 11 patients (68.8%) experienced complete pain relief, while 6 patients (37.5%) described their pain as mild and none reported severe pain. Among the 17 patients with preoperative limitations in ambulation, 6 (35.3%) achieved independent community ambulation without the need for assistive devices, and 10 (58.8%) continued to require such devices postoperatively. Notably, the one patient who was unable to ambulate in the community postoperatively died within three months following surgery. Functional outcomes were evaluated using the Musculoskeletal Tumor Society Score-93 (MSTS-93) system at the final follow-up, yielding a mean score of 18.9 ± 1.2 (range, 7-24) (**Table 3**).

**Table 2:** Preoperative characteristics of patients with metastatic disease of the acetabulum.

Patients	Gender	Age	Primary Tumor	Bone/visceral Metastasis	Previous Treatment	ASA Score	ECOG Status	Index Presentation	Harrington Type
1	M	66	Prostate cancer	Multiple/Lung	NO	3	4	Fracture	II
2	M	36	Penile cancer	Solitary/-	NO	2	3	Intractable pain	II
3	F	45	Lung cancer	Multiple/Mediastinal lymph node	NO	2	4	Impending Fracture	II
4	M	64	Bladder cancer	Multiple/Liver	Chemotherapy/Targeted therapy/Radiotherapy	3	4	Intractable pain	II
5	F	48	Gastrointestinal cancer	Multiple/brain	Chemotherapy	3	3	Intractable pain	II
6	F	41	Colon cancer	Multiple/Lung/abdominal lymph node	Chemotherapy/Targeted therapy	3	3	Impending Fracture	II
7	F	42	Lung cancer	Solitary/-	Chemotherapy	2	3	Fracture	III
8	F	53	Thyroid cancer	Multiple/-	Radiotherapy/Targeted therapy	2	3	Impending Fracture	II
9	M	44	Renal carcinoma	Solitary/-	Radiotherapy	3	3	Impending Fracture	III
10	F	44	Lung cancer	Multiple/brain	Radiotherapy/Targeted therapy	3	4	Impending Fracture	III
11	M	61	Renal carcinoma	Multiple/-	NO	2	3	Intractable pain	III
12	M	38	Lung cancer	Multiple/-	Chemotherapy	2	3	Intractable pain	III
13	M	61	Liver cancer	Multiple/-	Radiotherapy/Targeted therapy	3	4	Impending Fracture	III
14	M	56	Colon cancer	Multiple/Lung	Chemotherapy	2	4	Impending Fracture	III
15	F	56	Cervical cancer	Solitary/-	Chemotherapy	3	4	Impending Fracture	III
16	F	34	Lung cancer	Multiple/-	NO	2	3	Intractable pain	III
17	F	37	Cervical cancer	Multiple/Pelvic lymph nodes	NO	2	4	Impending Fracture	III

ASA, American Society of Anesthesiologists; ECOG, Eastern Cooperative Oncology Group.

**Table 3:** Intraoperative parameters and postoperative outcome of patients with metastatic disease of the acetabulum.

Patients	Gender	Age	Surgical time (h)	Blood loss (mL)	VAS score Pre/Post	Postoperative treatment	Complications	MSTS score	Follow up time (m)	Survival
1	M	66	2.4	500	7/3	Zoledronic Acid/ Endocrine therapy	None	7	3.9	Dead
2	M	36	3.2	300	7/2	Zoledronic Acid/CT/Immunotherapy	None	23	47.4	Alive
3	F	45	3.4	200	8/1	Zoledronic Acid/Targeted therapy	None	17	11.6	Dead
4	M	64	2.8	400	6/1	Zoledronic Acid/Targeted therapy	None	21	7.0	Dead
5	F	48	2.3	400	10/3	Zoledronic acid/CT	None	19	8.5	Dead
6	F	41	3.8	200	8/2	Denosumab/CT/Targeted therapy	None	19	9.7	Dead
7	F	42	3.3	300	9/2	Denosumab/Targeted therapy/RT	None	24	30.3	Alive
8	F	53	2.1	300	8/3	Denosumab/CT	None	16	15.1	Dead
9	M	44	3.7	600	9/4	Denosumab/CT/RT	Wound necrosis	21	16.6	Alive
10	F	44	2.4	300	9/3	Denosumab/CT/RT	Local recurrence	10	9.0	Dead
11	M	61	3.5	600	6/1	Denosumab/Targeted therapy	None	20	8.3	Alive
12	M	38	4.0	400	8/2	Denosumab/Targeted therapy	None	23	8.0	Alive
13	M	61	3.6	300	8/2	Denosumab/Targeted therapy/Immunotherapy	None	22	12.7	Alive
14	M	56	2.5	200	7/3	Denosumab/CT/Targeted therapy	Loosen	15	8.3	Dead
15	F	56	3.0	400	7/2	Denosumab/Targeted therapy	None	23	12.2	Alive
16	F	34	3.6	400	6/1	Denosumab/Targeted therapy	None	22	5.7	Alive
17	F	37	2.9	300	8/2	Denosumab/CT/Targeted therapy	None	20	4.4	Alive

VAS, visual analog scale; MSTS, musculoskeletal tumor society; CT, chemotherapy; RT, radiotherapy.

### 3.3 Survival and complications

**Figure 1:**
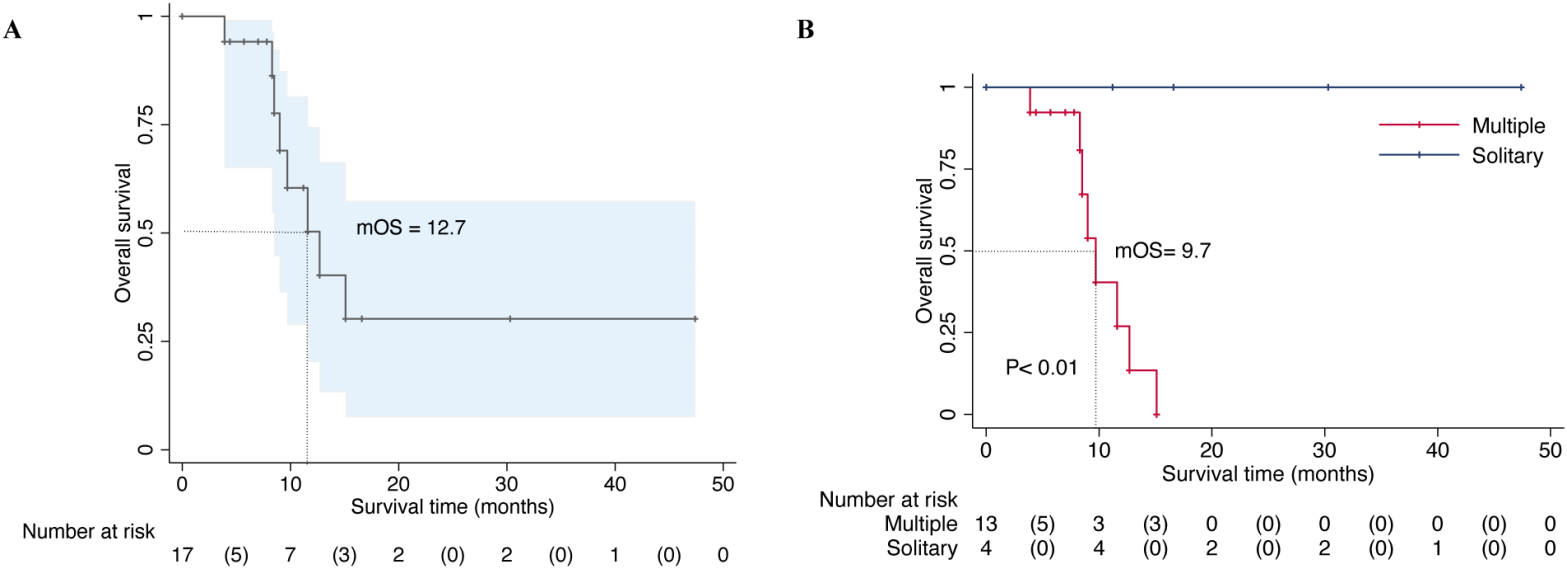
Overall survival of 17 patients with periacetabular metastasis who were surgically treated by microwave ablation combined with acetabular reconstruction **(A)**. Overall survival of patients based on the extent of the metastatic involvement (multiple or solitary metastases) and statistically significant differences between the groups were detected (P < 0.01) **(B)**.

**Figure 2:**
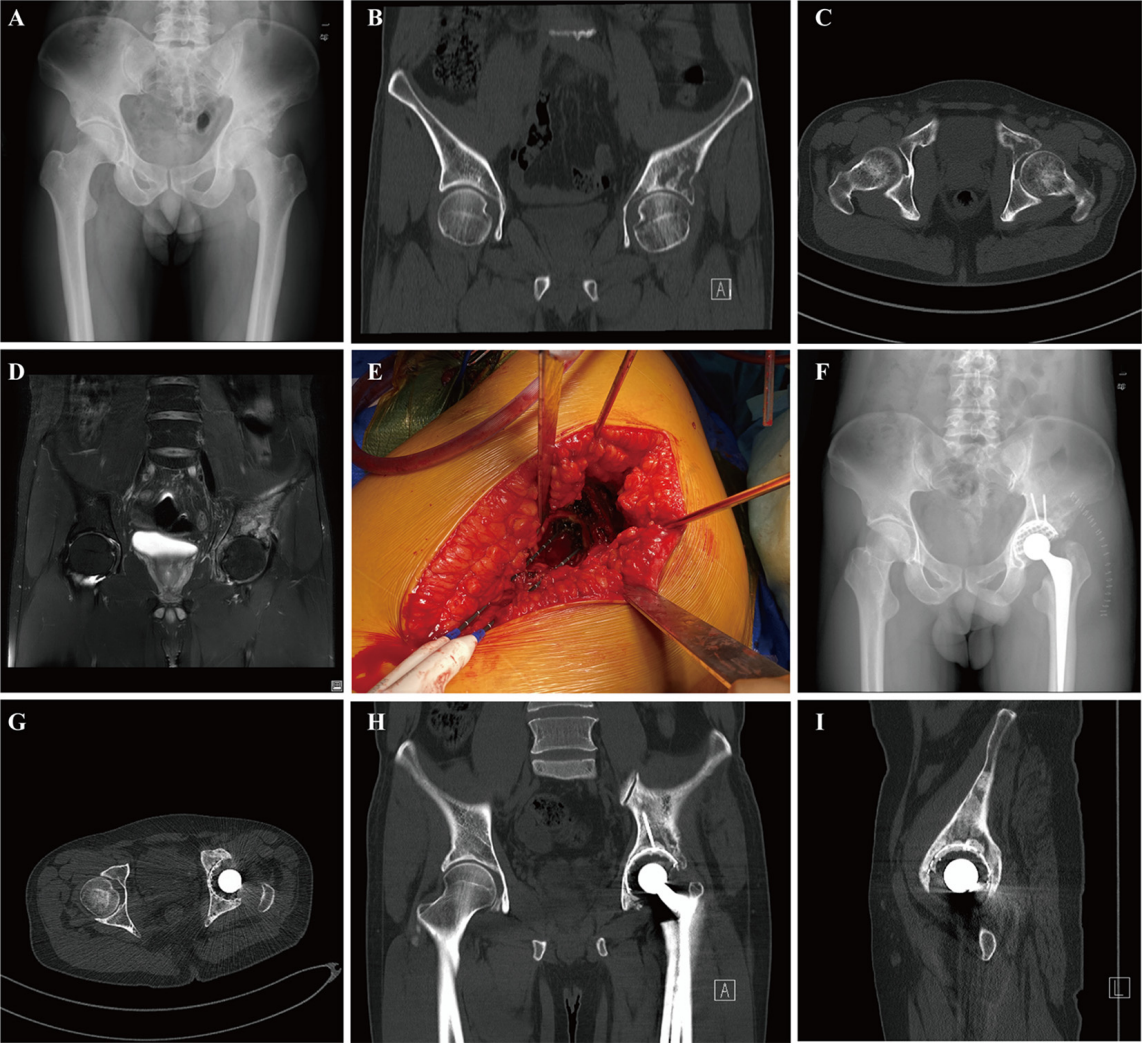
A 38-year-old male with multiple skeletal metastases from lung cancer who underwent microwave ablation combined with acetabular reconstruction: preoperative pelvic radiograph **(A)**, CT image (coronal **(B)** and axial **(C)**), and coronal MRI view **(D)** imaging of hip illustrating widespread lesions involving the acetabulum. Microwave ablation probes were inserted into the acetabular metastasis for ablation through the articular surface **(E)**. The postoperative radiograph showed the residual defect of the acetabulum was reconstructed by an acetabulum cage and cement total hip arthroplasty **(F)**. Postoperative 8 moths pelvic CT scans (axial **(G)**, coronal **(H)**, and sagittal **(I)**) demonstrated apparent osseous reconstitution and new bone formation within the defect at the surgical site of the periacetabular area without any sign of local recurrence.

In our series, all patients received postoperative systemic therapy, including chemotherapy, hormonal therapy, targeted therapy, or immunotherapy which were initiated 2 weeks to 1 month after the surgical treatment. Chemotherapy for 8 patients was delayed due to surgical wound healing and 3 patients received postoperative radiotherapy. All patients continued to receive bone-modifying agents (Denosumab) or bisphosphonates treatments to prevent bone-related adverse events. The median follow-up period was 9.0 months (95% CI: 7.8-12.6) in the last analysis, with a range from 3.9 to 47.4 months. Of the 17 patients, 9 patients remained alive at the last follow-up with a median overall survival of 12.7 months (**Figure 1A**). While the median survival of patients who had multiple metastases including bone and visceral lesions was only 9.7 months (**Figure 1B**). The estimated 12 months and 36 months overall survival of patients was at 40.9% (95% CI, 13.8-66.8) and 30.7% (95% CI, 7.8-57.7), respectively. During the follow-up period, 1 patient developed symptomatic loosening of the acetabular component at 6 months after surgery. The revision surgery involved extensive excision and modular hemipelvis reconstruction without change of femoral components. Superficial incisional necrosis occurred in 1 patient who received postoperative radiation therapy after acetabular reconstruction and MWA procedure. This patient was successfully treated with local debridement and systemic antibiotic therapy. Furthermore, 1 patient with lung cancer and multiple brain metastases developed local disease progression in the acetabulum at 8 months after surgery despite chemotherapy and targeted therapy. This patient underwent palliative radiotherapy for pain relief but declined further surgery and died 1 month later. In addition to the complications listed above, no patient encountered deep venous thrombosis, sciatic nerve palsy and heterotopic ossification. A representative case was a 38-year-old male with multiple skeletal metastases from lung cancer. After cage reconstruction combined with adjuvant MWA for the acetabular lesion, chemotherapy, and targeted therapy were administered. The follow-up radiograph demonstrated substantial new bone formation within the previously deficient periacetabular region (**Figure 2**).

## 4 Discussion

Recent advances in systemic therapy have contributed to more effective oncological treatments for patients with bone metastases, resulting in improved life expectancy and a heightened desire for a better-quality life. Nevertheless, a significant portion of patients continue to experience persistent, intractable pain, pathological fractures, and significant morbidity due to ongoing bone deterioration. As a result, despite these advancements, the management of periacetabular metastasis remains a complex challenge, with a lack of consensus on the optimal treatment strategy and considerable variability in the techniques employed.

Given the heterogeneity in acetabular metastasis, primary tumor type, and patient performance status, diverse surgical approaches have been adopted for acetabular reconstruction and to restore pelvic stability (24). Among these approaches, the classical technique introduced by Harrington and subsequent adaptations, offers patients prompt stability and pain alleviation, enabling unrestricted weight-bearing. While reasonable early clinical outcomes have been demonstrated, the Harrington procedures are linked to significant morbidity and a notable incidence of mechanical failure (25). In light of these challenges, alternative strategies such as porous tantalum implants, trabecular metal augments, saddle prostheses, and custom pelvic endoprostheses have been reported with varying success rates (6, 9, 16). Despite the range of techniques, the primary goal of surgery in patients with acetabular metastasis remains palliative, focusing on improving the quality of life. However, these complex interventions are often associated with increased complication rates and morbidity, especially in patients with limited physiological reserves (10, 26). Recent studies have highlighted the use of a specific antiprotrusio cage, which has shown satisfactory fixation and favorable short-term survival rates, with a low complication incidence (10, 27–29). Tsagozis et al. described the management of periacetabular metastasis by means of an acetabulum cage stabilized with retrograde screws and a cemented hip arthroplasty. The most common complication was dislocation occurring in 13 of 70 patients (19%), and overall survival of prosthesis was 92% at 1 year and 89% at 5 years, respectively (29). Rowell et al. reported the use of a stainless steel partial pelvic cage and cemented total hip arthroplasty for metastatic acetabular defects in 46 patients. Only one patient encountered radiological evidence of implant loosening and the reoperation rate was 8% at two years, primarily due to dislocation (10).

We attribute these improved results to several specific modifications. These include minimizing surgical trauma through a routine posterolateral hip approach with proximal extension. Moreover, the application of MWA has demonstrated significant pain relief through a synergistic effect that involves the destruction of nerve fibers within lesions, reduction of tumor burden, and decreased levels of nerve-stimulating cytokines (30). Intralesional curettage followed by the MWA procedure and subsequent reconstruction of the residual defect through cementation and arthroplasty components substantially reduced surgical complexity and operative time. Indeed, the mean surgical time with this technique was comparable to or shorter than that reported for other acetabular reconstruction methods (9, 25, 27). Additionally, by minimally dissecting the attachment of the gluteus medius in the ilium and using a constrained liner, none of the patients experienced perioperative infection or dislocation. Overall, 94.1% of patients in the present study experienced improvements in their ambulatory ability within three months postoperatively. Six patients could ambulate independently in the community, and ten patients used a single stick or walker. Moreover, the mean MSTS functional score was 18.9 (63%) in the surviving patients at the final follow-up, representing a significant improvement from preoperative status.

Hemorrhage is a significant risk during surgical interventions for periacetabular metastatic lesions. Preoperative selective arterial embolization of tumor vasculature is a widely accepted strategy for reducing intraoperative bleeding. However, its efficacy is inconsistent and difficult to predict preoperatively (31). Therefore, alternative intraoperative techniques to decrease bleeding are critical. Compared with other ablative methods, MWA is relatively insensitive to the intrinsic high impedance of bone, enabling deeper thermal penetration and greater power efficiency. This capability induces rapid coagulative necrosis of tumor cells and disrupts tumor vasculature (32). Moreover, MWA facilitates a higher ablation temperature within a shorter time and a wider ablation range by using multiple ablation probes (33). Our experience indicates that integrating MWA to manage acute intraoperative bleeding by charring the tumor and vessels prior to curettage is a valuable technique, especially for periacetabular metastases with extensive osteolytic lesions. In our study, the mean intraoperative hemorrhage was 358.8 mL, which is comparable to or less than that reported in studies employing modified Harrington procedures or megaprosthesis for acetabular reconstruction (16, 25). Although we do not have comparative data to definitively confirm the effectiveness of this technique, it appears that the current combination of preoperative arterial embolization and intraoperative MWA in patients with highly vascularized tumors could significantly decrease intraoperative hemorrhage.

Effective local tumor control is crucial for continuously improving quality of life and prolonging implant survival throughout the patient’s survival period (34). Previous studies have reported local progression rates of 25% to 35% with Harrington-style reconstruction or porous tantalum acetabular implants, which may result in mechanical failure of the reconstruction (9). Combined MWA procedure and decompression surgery for thoracolumbar metastases have been shown to achieve reasonable local tumor control, with no local recurrences observed (30). Similarly, a study of conservative surgery with MWA for recurrent bone tumors in the extremities reported reasonable local tumor control in all patients over a mean follow-up of 29.9 months (35). In the present study, we observed a low rate of local recurrence following intralesional curettage combined with adjuvant MWA. Specifically, within the limited survival time in this series, only one patient with multiple metastases experienced local recurrence, but this did not result in radiological loosening or implant failure. At the most recent follow-up, 9 patients remained alive while 8 patients had died, with median overall survival rates of 40.9% at 12 months and 30.7% at 36 months, respectively. Postoperatively, most patients showed evidence of new bone formation filling the defects at the surgical sites on follow-up radiographs. We attribute the increased osseous reconstitution to several factors: tumor destruction by MWA, the use of bone-modifying agents such as Denosumab or bisphosphonates, and effective advanced medical oncologic care.

The present study also has several potential limitations, the retrospective design and the lack of a comparison group may limit the generalizability of our findings. Moreover, the relatively small sample size and variability in bone defect types, primary tumor characteristics, and adjuvant therapy regimens might have affected patient outcomes. The follow-up duration was short for the majority of patients, often due to transfers to other facilities or disease progression resulting in death. The evaluation of local metastatic control was dependent on radiological assessments and patient-reported outcomes, which complicated the attribution of local tumor control to either surgical intervention or adjuvant therapy, particularly for tumors highly sensitive to radiation or systemic treatments. More studies with comparative data and larger patient cohorts are needed to further clarify the efficacy of these reconstruction techniques for periacetabular metastatic disease.

## 5 Conclusion

In conclusion, surgical treatment of metastatic disease around the acetabulum remains a significant challenge, particularly in a high-risk population. While extensive resection often necessitates complex reconstruction, the implementation of acetabular cages and cemented total hip arthroplasty presents a less invasive option. Moreover, the integration of microwave ablation with surgery may provide enduring pain relief, reduced intraoperative bleeding, enhanced local tumor control, and satisfactory functional outcomes postoperatively. However, to substantiate these results and refine treatment strategies, additional studies with more extensive patient cohorts and longer follow-up durations are crucial.

## Data Availability

The original contributions presented in the study are included in the article/Supplementary Material. Further inquiries can be directed to the corresponding author/s.
